# Mechanisms and Critical Technologies of Transport Inhibitor Agent (TIA) throughout C-S-H Nano-Channels

**DOI:** 10.3390/ma15020515

**Published:** 2022-01-10

**Authors:** Qi Luo, Jiale Huang

**Affiliations:** 1 School of Materials Science and Engineering, Southeast University, Nanjing 211189, China; qiluo@seu.edu.cn; 2 Jiangsu Key Laboratory for Construction Materials, Southeast University, Nanjing 211189, China; 3 State Key Laboratory of High Performance Civil Engineering Materials, Jiangsu Research Institute of Building Science Co., Nanjing 211103, China

**Keywords:** marine concrete, capillary adsorption, TIA, molecular dynamics, C-S-H nano-channels, hydrophobic, structure–activity relationship

## Abstract

The critical issue of the durability of marine concrete lies in the continuous penetration and rapid enrichment of corrosive ions. Here a new ion transfer inhibitor, as TIA, with calcium silicate hydrate (C-S-H) interfacial affinity and hydrophobicity is proposed through insights from molecular dynamics into the percolation behavior of the ion solution in C-S-H nano-channels and combined with molecular design concepts. One side of the TIA can be adsorbed on the surface of the cement matrix and can form clusters of corrosive ions to block the gel pores so as to resist the ion solution percolation process. Its other side is structured as a hydrophobic carbon chain, similar to a door hinge, which can stick to the matrix surface smoothly before the erosion solution is percolated. It can then change into a perpendicular chain shape to reduce the percolation channel’s diameter and thereby inhibit the percolation when ions meet the inhibitor. Therefore, once the erosion solution contacts TIA, it can quickly chelate with calcium ions and erosion ions at the interface to form clusters and compact pores. In addition, the water absorption, chloride migration coefficient, and chloride content of concrete samples decreased significantly after adding TIA, proving that TIA can effectively enhance the durability of cement-based materials. The structure–activity relationship of ion transfer that is proposed can provide new ideas for solving the critical problems of durability of cement-based materials and polymer molecular design.

## 1. Introduction

In recent years, marine concrete, as a significant member of marine infrastructure projects, has faced great challenges to its durability with the rise of marine infrastructure projects such as sea-crossing bridges and subsea tunnels [1,2,3,4,5]. The touch area between marine concrete and seawater can be divided into atmospheric areas, splash areas and underwater areas. The osculation of concrete and seawater can be simplified as wet–dry alternation in the splash zone. As the wave beats and dissipates, seawater invades and evaporates through the inner channel of marine concrete. Capillary channels are the most widely distributed channels in concrete, and their strong adsorption capacity enriches erosive ions quickly in capillary channels. In the underwater area, the concrete is immersed in seawater. With the continuous action of water pressure, erosive ions continue to penetrate through the internal channels of the concrete [1,4,5,6,7,8,9,10]. The main durability problem in marine concrete is corrosion of steel bars caused by penetration of corrosive ions through gel channels to the surface of the steel bars. As the main hydration product [11,12,13,14] C-S-H gel provides the percolation path for the eroding ions, which plays the main role in the strength of concrete. Its own composition is a porous structure, and it provides a percolation path for eroding ions. Regulating the percolation behavior of corrosive ions in C-S-H gel channels is of great importance for improving the durability of marine concrete structures.

Based on this understanding, adding hydrophobic materials or treating with corrosive ion complexes is an acceptable method to resist the intrusion of water or corrosive ions and thereby improve the durability of concrete [15,16,17,18,19,20]. At present, Liu has introduced a new nanomaterial for the cement hydration response, which can form insoluble nano-calcium carboxylate particles in parallel with the cement hydration process to regulate the gel channels of concrete [21]. Oltulu [22] found that nanomaterials can significantly improve the hardening and impermeability of cement mortar by regulating the microstructure volcanic ash reaction. Nanosilica and nanoalumina powder can synergistically improve the impermeability of mortar. Zhang [23] and Liu [24] examined the influence of nanosilica on the microstructure of the interfacial transition zone in high-performance concrete and found that nanosilica can reduce the area of the interfacial transition zone, improve the pore structure and significantly reduce chloride diffusivity. However, the application of nanomaterials in concrete has disadvantages, such as poor dispersion and difficulty of control. The liquid polymer can effectively avoid untoward dispersion in concrete. In addition, the polymer itself has a great degree of structural freedom and can achieve intelligent release and response under specific conditions according to the external environmental response, which has attracted much attention [25,26,27,28,29]. Many scholars have analyzed the influence of various polymers on the pore structure of concrete gel and adjusted the pore microstructure by polymers. Rixom [30] and Allyn [31] reported that olenyl-substituted succinic acid or oleic acid derivatives can react with calcium hydroxide produced by cement hydration and that insoluble particles will deposit in the pores of cement-based materials. Liu and Zhong analyzed the influence of styrene-acrylic emulsions on the permeability of concrete and found that polymers can form a continuous three-dimensional network structure in the cement matrix to improve chloride ion impermeability [32,33]. At present, most nanomaterials or polymers adjust the internal percolation paths of concrete structures to improve durability by regulating or regulating the microstructure of gel pores. However, a single durability improvement technology is challenging to meet the needs of high durability in the severe service environment of marine concrete. Therefore, efficient coupling hydrophobic suppression and dense hole plugging may be the key technologies to meet this demand. Mindess pointed out that the size of C-S-H gel pores is approximately 0.5–10 nm, and the percolation behavior of erosive solutions in cementitious materials is closely related to the size of gel pores [34]. However, the existing experimental resolution is limited to the micro- and nanoscales. It is necessary to study the in situ interaction on the molecular scale to inhibit the eroded solution’s percolation in gel capillary channels. In such a scenario, molecular simulations have been employed to reveal the structure [35,36,37,38] mechanical properties [39,40,41,42] and interfacial interaction of cement-based materials [43,44,45]. Meanwhile, molecular dynamics can also simulate the fluid percolation phenomenon in the C-S-H channel [46,47] and adjust the percolation rate of concrete pores by designing a corresponding transport inhibitor agent to prevent erosion ions from entering the concrete through the pores, further improving the durability of concrete and reducing the corrosion maintenance cost.

Hence, in this investigation, molecular dynamics were first employed to analyze the kinetic behavior of erosion solution percolation in a C-S-H matrix, and then an ion transport inhibitor was proposed according to two key technologies, called hydrophobic inhibition and hole blocking. After that, molecular simulation was used to gain insight into the regulatory effect of ion transfer inhibitors on the percolation behavior of erosion solutions. Finally, experiments were carried out to verify the effect and micromechanism of the ion transfer inhibitors.

## 2. Methods

### 2.1. Molecular Dynamics Methods

#### 2.1.1. Models

**Figure 1:**
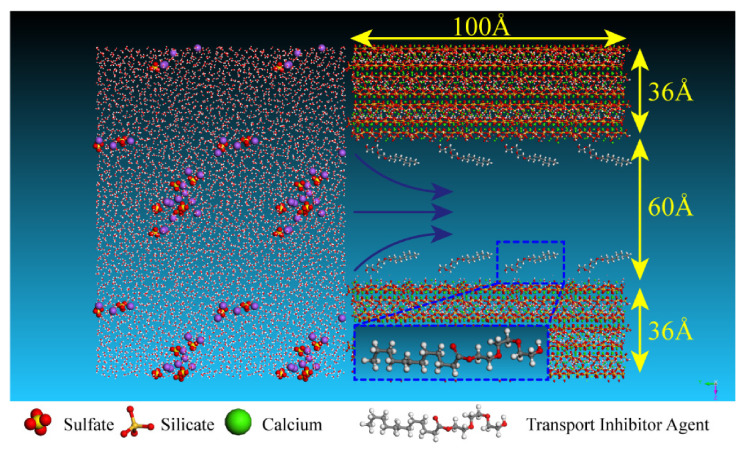
Schematic diagram of the calculation model (the left part is the erosive solution, the right part is the C-S-H gel channel, and the TIA polymers are distributed on the gel pore surface). The red, green, yellow, white, gray, purple and orange spheres represent oxygen atoms, calcium atoms, silicon atoms, hydrogen atoms, carbon atoms, sodium ions and sulfur atoms, respectively).

In the molecular model of the percolation process of aqueous solution/ionic solution in capillary pores, hydrated calcium silicate was used as the base of the hydration products, with a width of 5 nm and a length of 10 nm [34]. As described in [37,45] the calcium–silicon ratio was 1.3, in line with the range of calcium–silicon ratios of hydration products in marine concrete [48,49]. As shown in Figure 1, the left part was composed of aqueous solution and ions, in which the density of aqueous solution was 1 g/cm^3^, the concentration of NaCl solution 0.5 mol/L and the concentration of Na_2_SO_4_ 5 wt.%, all of which refer to the concentration range of ionic solution in the erosion area of marine concrete [50,51]. The right part was the C-S-H channel. A calcium silicate hydrate model with a length of 100 Å and width of 132 Å was constructed by cell expansion, then the pores with a width of 60 Å and a length of 100 Å were excavated in the middle of the model to construct the C-S-H channel in Figure 1. Driven by the capillary tension, the solution was to penetrate into the C-S-H nanopores, which is consistent with the solution erosion phenomenon in concrete [52]. TIA is a carboxylate polymer with one hydrophilic end and one hydrophobic end. It consists of 12 hydrophobic carbon chains and carboxylic acid groups, as shown in Figure 1 [21]. To simulate the actual conformation of TIA in concrete, the TIA was distributed according to the concentration of the pore solution measured by the experiment [21]. TIA has been proven to be a hydration-responsive additive. In the experimental test, TIA was adsorbed on the surface of the hydration products. Therefore, TIA was placed on the surface of C-S-H in the model to form an interlocking structure with 8 polymer segments. The total number of atoms in the model exceeded 44,555 to ensure its statistical significance on the atomic scale.

#### 2.1.2. Force Fields and Simulation Details

Clay force field (CLAYFF) and consistent valence force field (CVFF) hybrid force fields were used to describe organic–inorganic interactions. The CLAYFF force field has been verified to be applicable for the interfacial interaction of hydrated mineral systems with aqueous solutions [53,54,55,56]. The interaction between metal and oxygen was described by the LJ function and Coulomb term, and the SPC water model describes the behavior of water and hydroxyl groups based on a single point charge, which is beneficial for properly considering the capacity and momentum transfer between the liquid phase and solid phase. The CVFF force field is a uniform valence force field. It was originally suitable for calculating the molecular system of amino acids, water and various functional groups. After continuous strengthening, it can accurately predict the expansion, bending, dihedral angle distortion and off-plane vibration of organic molecular bonds [54,57]. In this paper, the force field parameters between atoms designed for organic–inorganic interactions were determined by the mean law. The distance parameters were calculated by means of the arithmetic mean, and the energy parameters were calculated by the geometric mean [58,59]. The model in this paper was established by the Material Studio 8.0 of Nanjing University’s High Performance Computing Platform Center. Lammps [60] was employed in molecular dynamics calculations, and periodic boundaries in the x, y and z directions were used for the model. First, the parameters of various atoms in the system were assigned by CLAYFF and CVFF hybrid force fields, in which the CLAYFF force field was used for the C-S-H msatrix and solution, and the CVFF force field for TIA. Calcium ions at the C-S-H boundary were then rigidly constrained to fix the gel pore position and relax the entire model’s energy. At the same time, the Nose–Hoover specification ensemble (NVT) was set at 298 K. In the model calculation, the rigid body was first fixed by setting a 3 Å area between the C-S-H and the etching solution. The solution area in the system was given a temperature of 300 K, and the rest of the structure was given an NVE ensemble. Then, a simulation of 1000 ps was performed. After the thermodynamic equilibrium between the polymer and the C-S-H interface in the C-S-H gel channel and the solution had been reached, the rigid restraint zone between the solution and the matrix was removed, and then a 300 K NVT ensemble was assigned to the system to simulate the percolation behavior of the solution under capillary adsorption of the gel channel. The system’s temperature, energy and pressure were monitored during the percolation process to prevent the system from destabilizing and to ensure rationality. Atomic displacement, velocity and trajectory were sampled every 0.1 ps for subsequent data processing of simulation results. This study used a 24-core CPU for calculations and takes of 102 h.

The radial distribution function represents the probability of seeking out atom B away from atom A at a distance r, calculated using Equation (1) [44].

(1) $$g_{AB(r)}=\langle N_{AB}(r)\rangle /\rho dV(r)$$

where $g_{AB(r)}$ refers to the probability of the B atom’s arising at *r* around the A atom; $\langle N_{AB}(r)\rangle$ is the ensemble average number of B atoms from *r* to *r* + *dr* around A atoms; ρ is the density of B atoms; and dV(r) is the volume of the spherical sphere shell from *r* to *r* + *dr* around A atom.

### 2.2. Experimental Methods

#### 2.2.1. Materials

The binding materials employed in this study mainly included cement, fly ash and slag, whose chemical composition is shown in Table 1. The cement was ordinary Portland cement PI 42.5 produced by China United Cement Co., Ltd., Beijing, China. Natural river sand with a fineness modulus of 2.6 (with an average particle size of 0.5–0.35 mm) and crushed stone with a water absorption rate of 1.3% were used as aggregates in this research. The polycarboxylic acid superplasticizer (PCE) is produced by Sobute Co., Ltd., Nanjing, China. It is designed based on special ionic functional groups and molecular rigid conformation, with space force and strong electrostatic repulsion. It can effectively improve the adsorption and dispersion capacity of the water reducer and its adaptability to concrete raw materials. It has the advantages of a water-reduction rate, plastic-preservation performance and high reinforcement. The polymer is a carboxylic ester polymer in the new TIA series developed by Sobute Co., Ltd. The preparation process of this kind of polymer is as follows: stir the organic solvent toluene and aliphatic organic carboxylic acid with 12 carbon atoms evenly, raise the temperature to 50~150 °C, add an appropriate amount of polyether and catalyst for reaction for 10~16 h to obtain the nanomaterial precursor. The mixing ratio of the concrete samples is shown in Table 2.

#### 2.2.2. Experimental Testing

The water absorption of concrete samples with and without ion transport inhibitors was tested according to the local standard of Jiangsu Province DB32/T 3696-2019. The chloride diffusivity of concrete was tested by a chloride diffusivity tester and concrete electric flux tester according to GB/T 50082-2009. Cylindrical concrete coated with epoxy resin was cut into 50 mm sheets and put into hydroxide solution to extract vacuum. The chloride permeability coefficient of three samples was tested according to ASTM C1202. In this experiment, the migration rate of chloride ion was measured by the electric acceleration method. First, put the sample into the voltage battery, use 0.3 mol/L NaOH solution for the positive electrode and 3% NaCl solution for the negative electrode. Then apply 60 ± 0.1 V DC to the sample for 6 h. The chloride migration coefficient was evaluated by a rapid chlorine migration (RCM) test system. The nonsteady-state migration coefficient was calculated by Equation (2) [61].

(2) $$D_{nssm}=\frac{0.0239\left(273+T\right)L}{(U-2)t}\left\{x_{d}-0.0238\sqrt{\frac{\left(273+T\right)Lx_{d}}{U-2}}\right\}$$

where

$D_{nssm}$ is the nonsteady-state migration coefficient (×10^−12^ m^2^/s);

T is the mean value of the initial and final temperatures in the test solution (°C);

L is the thickness of the test concrete specimen (mm);

U is the absolute value of the applied voltage (V);

t is the overall test time (h);

$x_{d}$ is the average chloride penetration depth (mm).

Two concrete samples were coated with epoxy resin in addition to the cut surface, saturated with water and finally immersed in 165 g/L NaCl solution. After 57 days, the specimens were carefully ground one by one perpendicular to the exposed surface. The total chloride concentration was obtained by titration based on standard NT Build 443. A Quanta 250 field emission scanning electron microscope (SEM) (FEI, Hillsboro, OA, USA) was employed to observe the morphologies of cement hydration products. The samples were coated with 15 nm gold before SEM testing.

## 3. Results and Discussion

As the essential component of the concrete matrix, C-S-H determines the overall strength and durability of concrete. Hence, C-S-H gel was employed as capillary matrix. The size of the percolation channel of the eroded liquid was the same as that of the C-S-H gel channel, both of which were 5 nm wide and 10 nm long. The hydrophobic substances generated by the hydrolysis of TIA under alkaline conditions were all around the nanopores. Therefore, to adapt to the actual situation, the TIA polymers are distributed on both sides of the pores in this model [18,21]. In this part, Section 3.1 and Section 3.2 describe the percolation kinetics of erosion solutions in C-S-H gel channels and the adsorption behavior of TIA by molecular dynamics. In Section 3.3, the working effectiveness of TIA was investigated by water adsorption test, chloride migration and micro morphology.

### 3.1. Percolation Kinetics of Erosion Solutions in C-S-H Gel Channels

**Figure 2:**
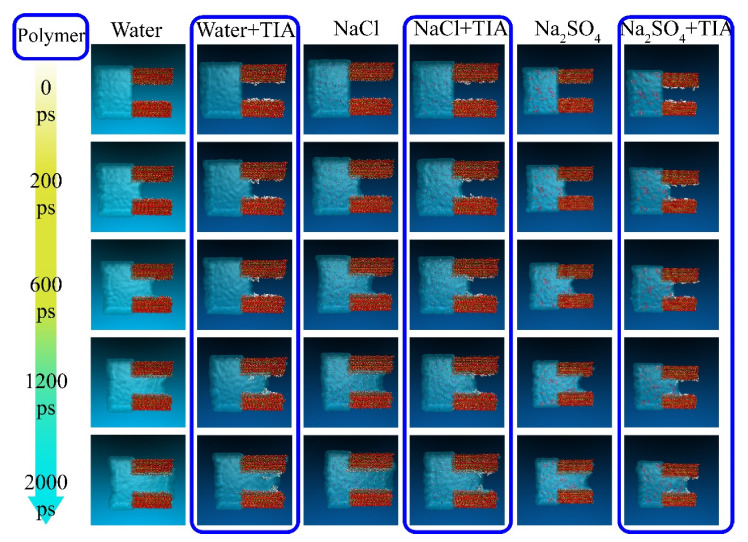
Percolation of various solutions in the C-S-H gel channel (the blue frame indicates that the C-S-H gel contains the TIA polymer). The red, green, yellow, white, gray, purple, bright green and orange spheres represent oxygen atoms, calcium atoms, silicon atoms, hydrogen atoms, carbon atoms, sodium ions, chloride ions and sulfur atoms, respectively).

**Figure 3:**
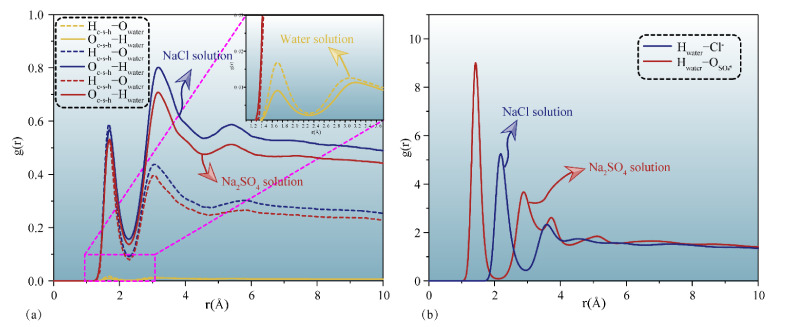
Radial distribution function between atoms from the solution and C-S-H matrix: (**a**) hydroxy from C-S-H versus hydroxy from different solutions, (**b**) hydrogen atoms from eroded solution versus eroded ions.

**Figure 4:**
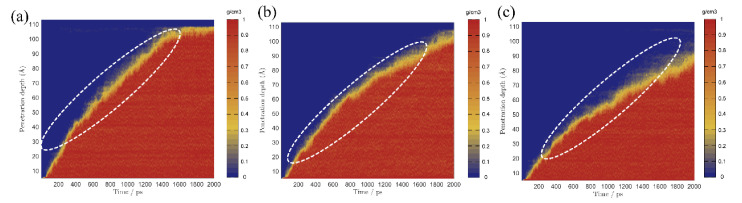
Percolation depth maps of different solutions in C-S-H gel channel without TIA: (**a**) manifests water solution, (**b**) demonstrates NaCl solution, (**c**) represents Na_2_SO_4_ solution.

**Figure 5:**
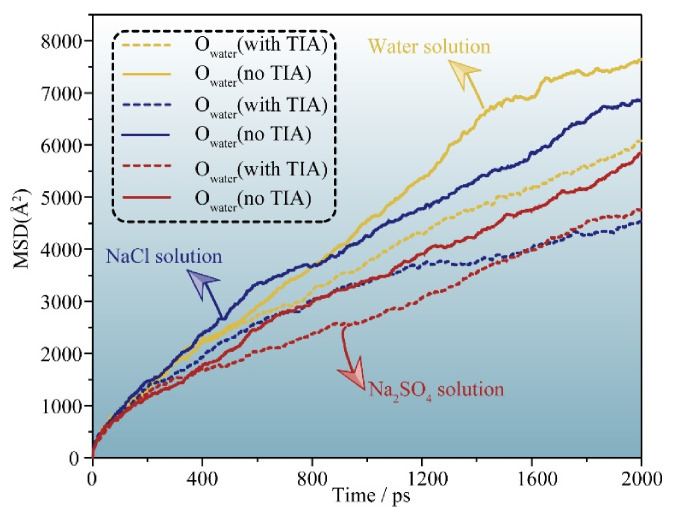
Mean square displacement function of oxygen atoms in water molecules in different solutions.

External liquids can permeate the gel pore through the negative capillary pressure due to the capillary permeation effect. Figure 2 displays a screenshot of the permeation process of different types of liquids into and out of the gel pore with or without TIA. As the simulation progresses, the solution gradually penetrates into the nanopore. It can be detected that the corrosive ions gradually penetrate the C-S-H gel pore along with the aqueous solution, while the forward direction of the solution shows that the upper and lower parts are fast, and the middle advances in the form of depression, indicating that the surface of C-S-H is hydrophilic. The radial distribution function in Figure 3a also implies this property. Both hydrogen atoms and oxygen atoms on the surface of the C-S-H matrix are bonded with oxygen atoms and hydrogen atoms of solution water, respectively. When corrosive ions exist, the hydrophilicity of the C-S-H interface seems to be enhanced, but Figure 4 represents that the percolation curve slope of pure aqueous solution is higher than that of corrosive solution, indicating that the percolation rate of pure aqueous solution is faster. This also implies that the percolation rate of pure aqueous solution is faster within the same time. Figure 4b,c demonstrates that the penetration depth curvature of the ionic solution system slows significantly from 800 ps. The percolation degree of aqueous solution in the pure water system reaches 90%, which is greater than that in the NaCl system and Na_2_SO_4_ system at 1200 ps. The same phenomenon can be reflected in the 1200 ps, as in Figure 2. The gel pores in the aqueous solutions are saturated, and the gel pores in sodium chloride solution and sodium sulfate solutions are also approximately saturated at 2000 ps. The mean square displacement function can provide insight into the kinetic information of atoms and molecules to reflect the percolation process of solution. Figure 5 illustrates the mean square displacement function of oxygen atoms in aqueous solution. The diffusion of oxygen atoms in different solutions is similar to that before 800 ps without adding an ion transport inhibitor. However, the diffusion ability of oxygen atoms in erosive solutions is weakened because of the positive charge at the interface of gel pores after 800 ps. The higher the negative charge of the erosive ions, the stronger the electrostatic adsorption on the interface with the gel pores. The sulfate ion charge in the sulfate solution is negative two, and the chloride ion charge in the chloride solution is negative, so that sulfate is more easily adsorbed onto the gel pore interface. The radial distribution function in Figure 3b shows that both sulfate and chloride ions have strong bonding with water molecules in solution, which also implies that the percolation rate of erosion solution is arranged as water solution > chloride solution > sulfate solution. Therefore, the percolation of aqueous solution and the adsorption of corrosive ions on the gel–pore interface should be considered to mitigate the erosion of seawater in marine concrete engineering. Design criteria for ion transfer inhibitors can be obtained by analyzing the percolation behavior of corrosive solutions in gel voids and critical techniques to solve the durability of marine concrete: (1) TIA needs to prevent the penetration of corrosive solutions into gel channels effectively; (2) TIA can respond quickly and react with the corrosive ions to form a complex product that settles on the surface of the pore and then densifies the pore once the corrosive ions penetrate the gel pore.

### 3.2. Adsorption Behavior of TIA in C-S-H Gel Channels

**Figure 6:**
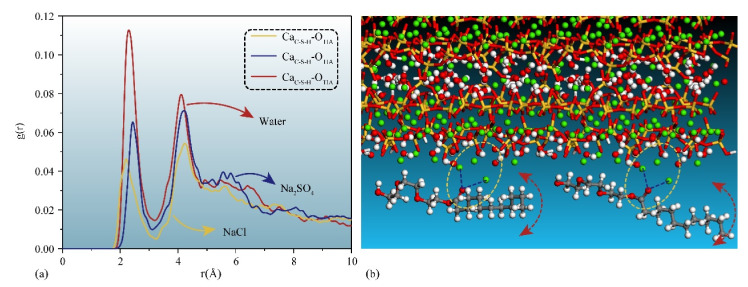
Interaction between TIA and C-S-H matrix surface in different solutions: (**a**) represents radial distribution functions of oxygen atoms in TIA and calcium ions on the surface of C-S-H matrix in different solutions, (**b**) indicates the binding of TIA to calcium ion. The red, green, yellow, white and gray spheres represent oxygen atoms, calcium atoms, silicon atoms, hydrogen atoms and carbon atoms, respectively.

TIA demonstrates affinity on both sides of the C-S-H pore before the solution penetrates the C-S-H gel pore. As shown in Figure 2, TIA aggregates on the C-S-H surface, and their conformations are clustered or crooked at 0 ps. In different solution systems, the TIA polymer adsorbs to the C-S-H matrix. As shown in Figure 6a, the radial distribution function was employed to observe the source of its adsorptive force. The polar functional group -COO- on the polymer coordinates with calcium on the surface of the C-S-H matrix, and adsorption is not limited by the solution system. The peak value of the radial distribution function was approximately a distance of 2.3~2.5. Figure 6b indicates that calcium ions and oxygen atoms exist stably at the interface. Previous studies have also confirmed that the polar functional group COO- of polar polymers can interact strongly with the calcium ions of C-S-H 45,65,66. However, Figure 2 shows that the connection between the other end of the TIA and the C-S-H matrix is not close at 0 ps. In the Naso/TIA system, it is found that the end of the carbon chain of TIA is far away from the C-S-H interface. At this time, the conformation of TIA constitutes an interesting phenomenon: one end of the polymer is fixed on the C-S-H surface, and the other end is reliable near the C-S-H surface and far away from the C-S-H surface, which is similar to a closure page on a door.

### 3.3. Inhibitory Transport Kinetics of TIA in C-S-H Gel Channels

**Figure 7:**
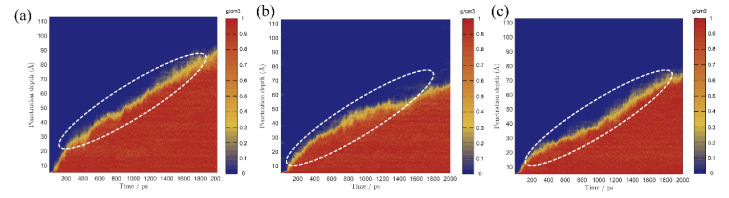
Percolation depth maps of different solutions in C-S-H gel channel with TIA: (**a**) manifests water solution, (**b**) demonstrates NaCl solution, (**c**) represents Na_2_SO_4_ solution.

**Figure 8:**
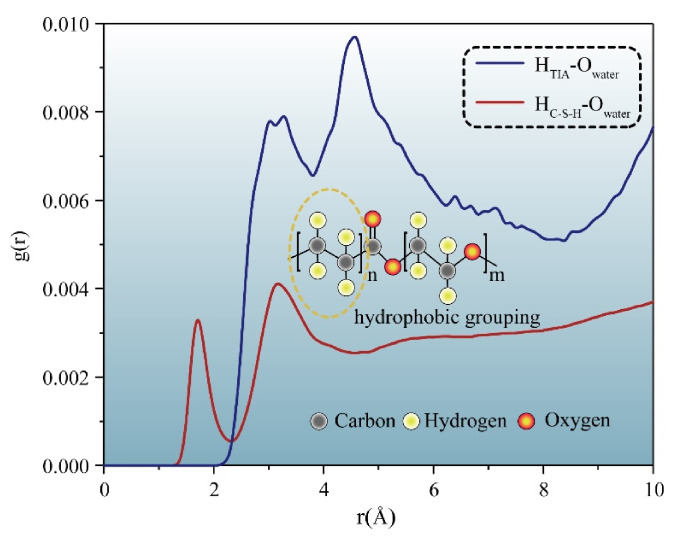
Radial distribution function of oxygen atoms in solution and hydrogen atoms in the system.

To compare the permeation processes of three types of solutions with/without TIA (water, NaCl, Na_2_SO_4_), a function of the penetration depth and time of the solution in the gel channel is recorded in Figure 7. In the first 200 ps, when the solution does not penetrate the position of the TIA, the penetration depth of the same solution with or without the TIA is similar and almost coincides. The solution begins to contact the TIA at 200 ps. However, Figure 7 demonstrates that the percolation depth gradient decreases sharply after 200 ps, suggesting that the percolation rate decreases sharply, which results in a larger gap between the percolation depths with and without the TIA. Figure 2 illustrates that the solution initially contacts the TIA at 200 ps, and the conformation of the TIA changes from lying flat on the C-S-H surface to vertically, almost flush with the solution surface. These fluid transfer inhibitors are attributed to the hydrophobic carbon chain of TIA. The source of the hydrophobic effect can be observed from the radial distribution function in Figure 8. It can be found that oxygen in solution is more likely to form hydrogen bonds with C-S-H, which is opposite in TIA comparing hydrogen atoms on the surface of C-S-H with hydrogen atoms on the hydrophobic chain of TIA. Therefore, when the TIA on both sides of the gel pore contacts the aqueous solution, the conformation of the TIA changes to a vertical state, which reduces the width of the solution percolation pore channel and the percolation rate of the solution due to the action of hydrophobic carbon chains.

In the presence of TIA, Figure 2 demonstrates that the forward direction of each solution is almost perpendicular to the C-S-H interface, in sharp contrast to that without TIA, because TIA weakens the hydrophilicity of the C-S-H surface and behaves as a hydrophobic phenomenon. This phenomenon is related to the conformational changes of TIA at the 0 ps. TIA achieves capillary hydrophobic effects by improving the hydrophobicity of gel pore channels. In addition, with the percolation of the solution, the TIA aggregates at the front end of the solution. Figure 7 demonstrates that the curvature of the penetration depth begins to slow down at 800 ps in the presence of TIA. Notably, the presence of TIA in the NaCl and Na_2_SO_4_ solutions makes the overall percolation process slower than in aqueous solutions, on account of the interaction of erosion ions with the substrate surface and TIA polymers.

**Figure 9:**
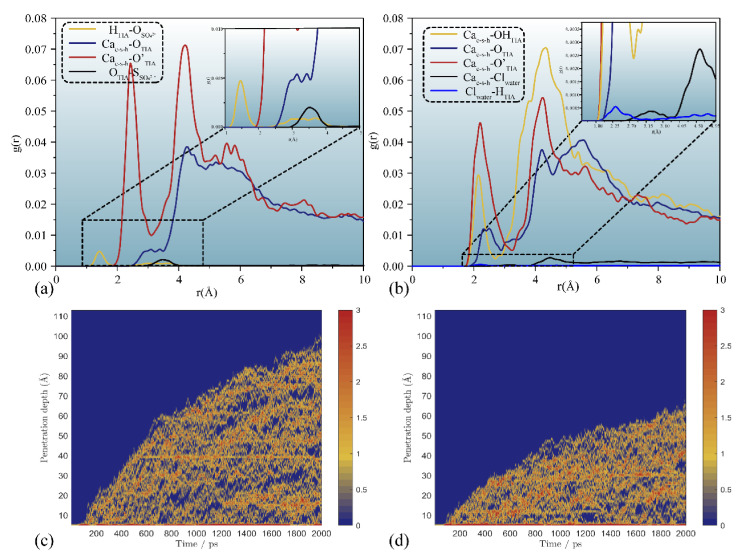
Radial distribution function among erosion medium, matrix and polymer atoms in the percolation process of (**a**) sodium sulfate solution and (**b**) sodium chloride solution and chloride ion percolation depth diagram in sodium chloride solution (**c**) without TIA (**d**) with TIA.

Figure 2 and Figure 7 illustrate that the percolation depth of the NaCl solution is similar to that of the Na_2_SO_4_ solution, but there are differences in the percolation process. Figure 7b,c demonstrates that the rise rate of the percolation depth curve of the Na_2_SO_4_ solution is slower than that of the NaCl solution. The radial distribution function of Figure 9a implies that the H atom of TIA bonds firmly with the O atom in sulfate with a radius of 1.5 Å. In addition, the Ca ion on the C-S-H surface bonds strongly with TIA. Sulfate ions can chelate with TIA and C-S-H, produce cluster structures, and ultimately block the percolation pores. A similar chelation effect was also observed in the NaCl solution. The radial distribution function of Figure 9b presents that there is bonding between the H atom of TIA and Cl ions at 2.25 Å. Hence, the chelation effect of Cl ions with TIA and C-S-H is formed. However, the bonding action of Cl ions is less than that of sulfate ions, so that the clusters produced in Na_2_SO_4_ solution are more stable than those produced in NaCl solution.

As shown in Figure 9c,d for the chloride ion penetration depth, chloride ions can almost penetrate the C-S-H gel pore without TIA addition. However, after TIA addition, the penetration depth of chloride ions reaches only half the length of the gel pores, and the model system tends to be balanced. This shows that TIA can effectively reduce the penetration depth of chloride ions. Mainly due to the two mechanisms mentioned before, (1) the radius of gel voids decreases; and (2) calcium ions, erosion ions and TIA chelate to form clusters, which densify gel pores. In summary, molecular dynamics simulations show that TIA inhibits the percolation behavior of different erosion solutions in C-S-H gel pores. The mechanism by which TIA inhibits transmission is mainly hydrophobic inhibition and dense pores. This forms the structure–activity relationship of ion transfer inhibitors and provides theoretical guidance for the molecular design of ion transfer inhibitors.

### 3.4. Durability Test Verification

**Figure 10:**
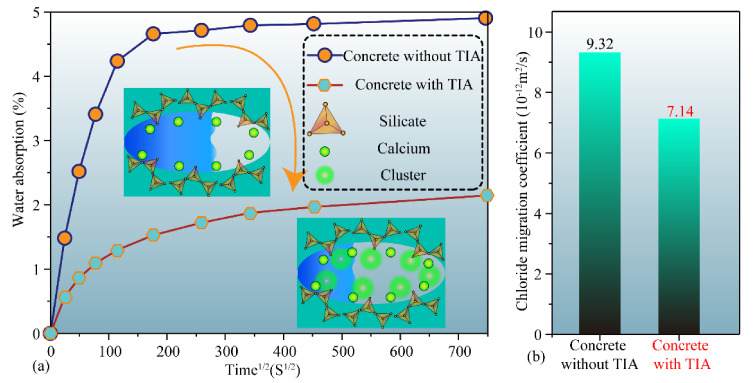
Water absorption and diffusion coefficients of concrete with and without TIA (**a**): the water adsorption ratio of cement pastes as a function of immersing time; (**b**) the chloride migration coeffient of concrete with and without TIA.

Figure 10a illustrates the water absorption results of concrete samples without TIA and with TIA. The results show that the water absorption ratio increases as percolation proceeds for each group of concrete mixtures, but the water absorption rate of concrete samples with TIA is lower than that of samples without TIA. Samples without TIA reach the approximate saturation state of percolation at 200^2^ s, while samples with TIA do not reach saturation at 350^2^ s. The water absorption ratio of the samples without TIA is almost 5%, while that of the samples with TIA is only 2% at 750^2^ s. This hints that TIA can effectively restrain the rate of the water absorption ratio of concrete and reduce the water absorption of concrete, thus improving the medium impermeability of concrete.

**Figure 11:**
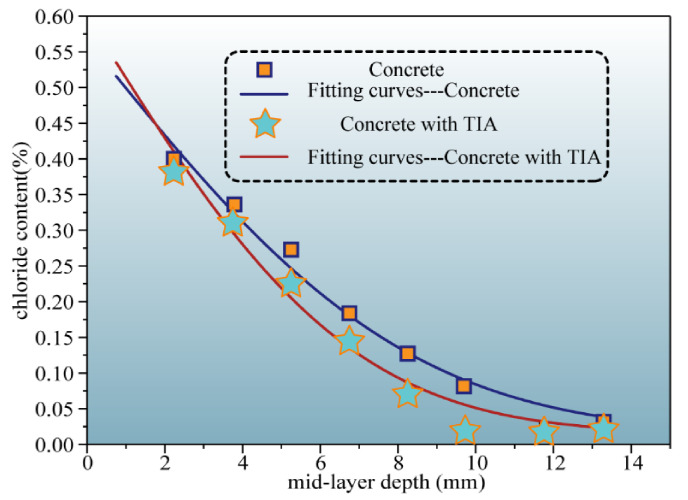
Chloride content of concrete and its fitting curves.

**Figure 12:**
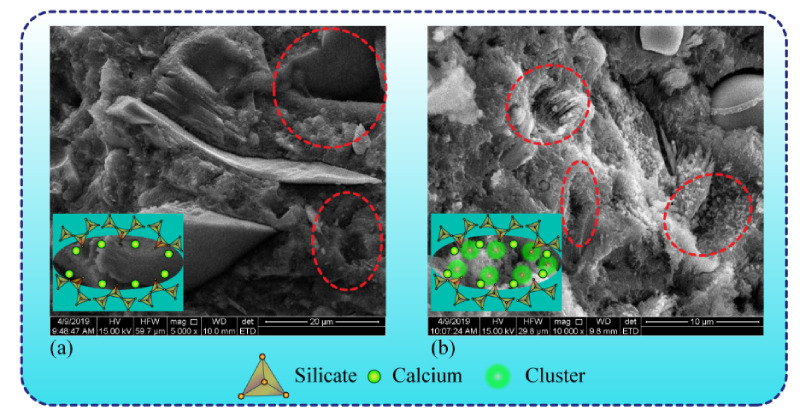
SEM micrograph of cement pastes (**a**) without and (**b**) with TIA after 28 days of hydration time.

The chloride diffusion coefficient and surface chloride concentration were calculated by fitting the relevant data points into Equation (2) by means of nonlinear minimum regression analysis. The chloride migration coefficient more directly reflects the resistance of concrete to ion erosion. Figure 10b manifests that the chloride migration rate of samples without TIA reaches 9.32 × 10^−12^ m^2^/s, while the incorporation of TIA reduces the chloride migration rate to 7.14^−12^ m^2^/s. This means that TIA can effectively slow down the percolation of chloride ions in the concrete pores, which corresponds to the molecular dynamics simulation results shown in Figure 9c,d. The test results for the surface chloride content are shown in Figure 11. They demonstrate that the surface chloride content of concrete samples with and without TIA decreases gradually with test depth. Evidently, the difference between the surface chloride content with TIA and that without TIA gradually increases as the test depth increases. This implies that the presence of TIA can effectively reduce the chloride content of samples. This phenomenon is mainly due to the hydrophobic properties of TIA. The microscopic morphology of the two types of samples was observed to explain this phenomenon. As shown in Figure 12, there are many microcluster structures in the microvoids of the sample surface after doping with TIA. In addition, the molecular simulation results indicate that TIA can chelate with calcium ions in gel pores and generate clusters to block the pore channels, thus impeding the penetration of solution. We must admit that SEM cannot demonstrate the in situ morphological changes caused by organic modification due to limitations in resolution. However, SEM can display the morphology difference of cement paste after 28 days of hydration with/without TIA, which is helpful for thinking about the possible mechanism of TIA. It is worth noting that this is only an auxiliary means. In other words, the microscopic test method provides the characterization results of cement paste mixed with TIA and cannot show the actual hydration process. Nevertheless, molecular dynamics can show the conformational changes of TIA on the surface of cement hydration products and the in situ interface interaction mechanism. The combination of these two methods is helpful to understand the possible mechanism of TIA. In conclusion, TIA can not only alleviate the percolation of the solution medium by hydrophobic action, but also chelate with calcium ions in C-S-H gel pores to form clusters. When the eroding solution is percolated, TIA, Ca^2+^ and eroding ions can induce a stronger chelating effect, which is more likely to result in clusters blocking the gel pore channels.

## 4. Conclusions

The properties of cement-based materials are mainly affected by dry–wet alternations and long-term immersion in marine engineering. Capillary adsorption and the sustained action of water pressure promote the penetration of corrosive solution from seawater into cement-based materials, which injures the matrix and the reinforcing bars in the cement-based materials. To solve this problem, a new type of TIA for cement-based materials was designed, manufactured and tested.

1. Molecular dynamics unravel the interaction mechanisms between TIA and the C-S-H gel matrix. TIA has hydrophilic and hydrophobic groups, which are linked by carboxyl groups.
2. The structure–activity relationship contributes to the design of TIA. Hydrophilic groups in TIA can effectively adsorb to the surface of the C-S-H matrix by bonding with calcium ions, while hydrophobic carbon chains on the other end can float on the solution surface through low-hydrogen bonding when the solution is in contact with it; thus, the effective diameter of the gel pore can be reduced, and the solution penetration can be prevented with the ‘door’ of the gel pore.
3. In corrosive solution, Ca^2+^ in C-S-H chelates with TIA and corrosive ions, forming clusters to block gel pore channels, making the blocking effect more obvious. The experimental test results manifest that the water adsorption capacity, chloride ion migration rate, and chloride content of the concrete samples reduced significantly after adding TIA, indicating that TIA can effectively strengthen the durability of concrete.

## Figures and Tables

**Table 1:** Chemical composition of binding material (mass %).

Oxide	Cement	Fly Ash	Slag
CaO	61.75	7.40	41.5
SiO_2_	20.64	43.9	32.2
Al_2_O_3_	4.62	34.8	14.6
Fe_2_O_3_	2.82	6.13	0.96
K_2_O	0.48	1.09	0.57
MgO	2.06	0.65	6.37
Na_2_O	0.12	0.43	0.30
SO_3_	1.20	2.00	2.12
TiO_2_	0.29	1.51	0.61

**Table 2:** Mix design of concrete (kg/m^3^).

Sample	Cement	Slag	Fly Ash	Water	Sand	Fine Aggregate	Coarse Aggregate	PCE	TIA
0.4	269.5	147	73.5	196	702.74	404.504	606.756	0.8‰	0%
28 L	7.546	4.116	2.058	5.444	19.677	11.326	16.989	54.88	0 g
0.4	269.5	147	73.5	196	702.74	404.504	606.756	0.8‰	0.90%
28 L	7.546	4.116	2.058	4.882	19.677	11.326	16.989	54.88	686 g

## Data Availability

Not applicable.
